# Effects of Rest Time and Curing Regime on Short- and Long-Term Strength of Class C Fly Ash-Based Alkali-Activated Mortars

**DOI:** 10.3390/ma17071632

**Published:** 2024-04-02

**Authors:** Cedric Kashosi, Ahmed Gheni, Eslam Gomaa, Mohamed ElGawady

**Affiliations:** 1 Department of Civil, Architectural, and Environmental Engineering, Missouri University of Science and Technology, Rolla, MO 65409, USA; cckgbc@umsystem.edu (C.K.); eyg9qc@umsystem.edu (E.G.); 2 Civil Engineering Department, Komar University of Science and Technology, Sulaymaniyah 46001, Iraq; ahmed.gheni@komar.edu.iq; 3 Department of Structural Engineering, Faculty of Engineering, Cairo University, Giza 12613, Egypt; 4 Walker Consultants, New York City, NY 10018, USA

**Keywords:** class C FA, alkali-activated mortar, rest time, ambient curing, oven curing, steam curing, long-term strength development

## Abstract

This study investigated how different rest times affect the strength development of fly-ash-based alkali-activated mortar (AAM) over a period of 90 days. Two types of fly ash with varying calcium oxide contents of 37 and 21% were used. The rest times ranged from 2 to 36 h, and three curing methods (ambient, oven, and steam) were tested. The results showed that the rest time significantly influenced the compressive strength of the AAM. The optimal rest time was found to be between 12 and 30 h depending on the curing method and fly ash type. Beyond this range, there were only minor changes in strength. One type of fly ash (FA21) showed higher strength with longer rest times up to 30 h, while the other type (FA37) had the highest strength within a rest time range of from 12 to 24 h. Over the 90-day period, the specimens cured under ambient, oven, and steam conditions at 55 °C (131 °F) experienced increasing strength, but those steam-cured at 80 °C (176 °F) showed a decrease in strength. Analysis revealed the formation of hydration products in FA37, while FA21 showed a reduction in peaks for its main compounds. Additionally, XRD analysis revealed the formation of hydration products (CSH and CASH) in FA37, while FA21 displayed a reduction in peaks for its main compounds. EDS analysis indicated the presence of partially unreacted FA particles, highlighting the impact of curing methods on dissolving FA particles and the formation of geopolymer products (NASH and CNASH) responsible for compressive strength development.

## 1. Introduction

Alkali-activated concrete (AAC) is an emerging eco-friendly material with many performance and sustainability advantages [1,2,3]. While there has been extensive research on class-F-fly-ash-based AAC, much less research has been carried out on class-C-fly-ash-based AAC. Therefore, there remain many questions related to the preparation process and its impact on both short- and long-term behavior. The rest time, which is the time between the end of the mixing process and the beginning of the heat curing, is an important parameter in the process of producing AAC that has an influence on both the strength and durability of AAC. 

Studies showed that increasing the rest time up to 24 h improved the strength of AAC [4,5]. Applying a rest time of 24 h to alkali-activated class F FA (AAFFA) pastes having a calcium content of 3.0% before curing at 50 °C (131 °F), 65 °C (149 °F), or 80 °C (176 °F) for two days resulted in about a 50% increase in the compressive strength compared to curing for three days at those elevated temperatures without applying any rest time [5]. Furthermore, a rest time of 24 h for AAFFA pastes with a calcium content of 6.5% followed by curing at 75 °C (167 °F) for 24 h was beneficial for the strength compared with no rest time [4]. On the positive hand, the rest time allows for the dissolving of silica and alumina species to form an aluminosilicate gel, leading to a higher strength. Also, the rest time prevents cracking due to an abrupt loss of moisture [6,7].

Increasing the rest time beyond a certain period of time does not have an effect on the strength of AAC [8] or is in fact harmful for the strength of AAC [9]. In the case of AAFFA mortar with a calcium content of 16.6%, a rest time of one hour before curing in an oven at 60 °C (140 °F) increased the strength by 16% compared to no rest time [9]. However, increasing the rest time beyond that for either three or six hours slightly decreased the strength by approximately 8% [9]. Furthermore, there was a very little difference between the strengths of two sets of specimens cured at 60 °C (140 °F) with and without a rest time of one hour [8]. 

The literature lacks consensus on the optimal rest time for AAC (alkali-activated concrete) and whether longer rest times enhance its strength. Previous studies have used varying rest periods ranging from zero to 24 h [6,10,11,12,13,14,15,16] with no consistent pattern. Moreover, there is a scarcity of studies exploring the optimization of the rest time specifically for alkali-activated class C FA (AACFA). Therefore, further research is necessary to determine the rest time that yields optimal strength for AACFA.

Another important consideration for AAB (alkali-activated binder) is long-term strength development, which is influenced by the curing temperature and regime. In traditional OPC (ordinary Portland cement) concrete, the majority of the strength is attained after 28 days of curing at ambient temperature. Since AAC is a novel material that may require elevated-temperature curing in certain cases, it is crucial to study the strength development of AAM (alkali-activated material) beyond the 28-day mark using various curing temperatures.

The development of the properties of AAFFAs cured at 80 °C for 24 h has been investigated up to an age of 365 days [17]. Although the specimens were cured at an elevated temperature, an average strength development of 23.2% was observed between 28 and 365 days. However, in another study, the average strength development was 6.6% of the AAC synthesized with class F FA that was observed between 28 and 56 days. This is different from that of ground granulated blast furnace slag (GGBFS), where the strength of GGBFS binders increased up to 90 days, after which a strength degradation was observed due to the combined effect of the disjoining pressure and self-desiccation, resulting in a less dense CSH gel [18]. Furthermore, the compressive strength of AAFFAs cured at 60 °C (140 °F) for 24 h increased by 10% between 7 and 28 days and was approximately constant between 28 days and 1095 days [19]. For those that were ambiently cured, the strength development was approximately 125% between 7 and 84 days [19].

Using a steam curing regime at an elevated temperature is common practice in the precast industry as it yields an early high strength for conventional concrete [20]. The use of a steam curing regime to produce AAB synthesized using slag [21,22,23] and class F FA [24] was successfully applied. However, when the steam and dry-oven curing regimes were applied to the AAB synthesized using class F FA, the results showed that the steam curing had a negative effect on the compressive strength compared to those cured in dry conditions [19,25]. The compressive strength of AAFFAs cured at 60 °C (140 °F) for 24 h and at 95 °C (203 °F) for 8 h without steam was higher than that of those cured with steam with an average of 15% [19] and 28% [25]. The effect of applying a steam curing regime on the short- and long-term strength of AACFA is not well documented and needs further study.

This study investigated the rest time and the long-term strength and temporal evolution of AACFA mortar cured under different regimes. The specimens were subjected to rest times ranging from 2 h to 36 h prior to being cured to determine the optimum rest time. Once the optimum rest time was determined, the specimens were cured under ambient, oven, or steam conditions. The steam and oven curing were carried out for nine hours at two different temperatures of 55 °C (131 °F) or 80 °C (176 °F). The mortar specimens were then tested for their compressive strength at different ages up to 90 days. Also, the microstructures of alkali-activated paste (AAP) mixtures were examined in order to better understand the performance of the different mixtures. 

## 2. Research Significance

Only limited research has focused on class-C-fly-ash-based alkali-activated binders, primarily examining their short-term behavior and neglecting long-term strength development. Additionally, while steam curing at elevated temperatures is commonly used in the precasting industry to achieve early high strength in conventional concrete, there is a lack of research on the performance of steam-cured alkali-activated class C fly ash binders. Moreover, the existing literature predominantly employs a standard rest time of two hours, but unlike alkali-activated class F fly ash (AAFFA), there is a dearth of detailed research into the influence of the rest time on the strength of alkali-activated class C fly ash (AACFA). This manuscript addresses these gaps by exploring the effects of the rest time and curing regimes on the strength of AACFA mortar and investigating its strength development up to 90 days.

## 3. Experimental Program

**Figure 1:**
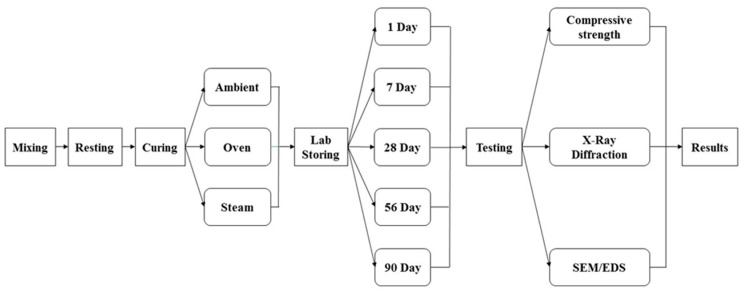
Schematic overview of the work.

Figure 1 illustrates a schematic overview of the whole experimental work of this paper, and the following sections explain the details of each step.

### 3.1. Material Characteristics

#### 3.1.1. Fly Ashes

Two FAs, classified as class C as per ASTM C618-19 [26,27], were used as precursors (Table 1). The nomenclature of each FA consisted of the letters “FA” followed by a two-digit integer representing the calcium oxide (CaO) content in the FA. For example, FA37 had a CaO content of approximately 37%.

#### 3.1.2. Alkali Activators

Two solutions, sodium hydroxide (SH) and sodium silicate (SS), were used as the alkali activators. A 10 M SH solution was prepared by mixing solid SH pellets with distilled water.

### 3.2. Mixtures Proportions and Preparation

#### Mix Design

Four alkali-activated mortar (AAM) mixtures were prepared in this study using FA37 and FA21 with two different mix designs: HA and HS (Table 2). The alkali-activator-to-FA (Alk/FA) ratios were selected based on an optimization study [28]. The mixtures were prepared by following the mixing procedure outlined in [29,30].

### 3.3. Fresh Properties of the AAM

The workability and initial setting time of the AAM mixtures were measured using the flow table as per ASTM C230-14 [31] and a modified Vicat needle as per ASTM C807-13 [32].

### 3.4. Casting, Curing Regimes, and Rest Time

The fresh AAM was cast into 50 mm (2 in) cube molds as per ASTM 109-16 [33]. Three curing regimes, ambient, steam, and oven, were applied to the AAM mixtures. For the ambient curing method, the mortar specimens were kept in their respective molds and covered with an impermeable sheet to minimize moisture loss. Then, the specimens were placed in the laboratory at an ambient temperature of 23 ± 2 °C (73 ± 3 °F) until the required testing age. 

**Figure 2:**
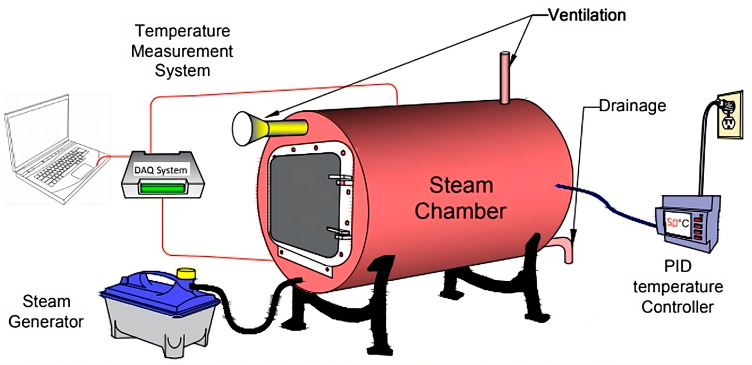
Steam chamber system.

For both the steam and oven curing, the specimens had rest time periods of either 2 h, 6 h, 12 h, 24 h, 30 h, or 36 h before starting the curing process [34]. During the rest time, the specimens were cured at ambient temperature, as explained earlier, where the specimens were allowed to set and develop an initial strength that minimized the number of microcracks during the elevated-temperature curing. For the steam curing, the specimens were subjected to a combined heated and humid environment created inside a steam chamber at two different temperatures of either 55 °C (131 °F) or 80 °C (176 °F) for 9 h (Figure 2). The 9 h period was found to result in the highest strength. For the oven curing, the AAM specimens were encased in thermal bags and placed in an oven at two different temperatures of either 55 °C (131 °F) or 80 °C (176 °F) for 9 h. Thermal bags were used to avoid loss of moisture.

At the end of the required oven and steam curing time, the cured specimens were removed from their respective molds and put in the moisture room until either the test day or for seven days, whichever occurred first; then, the specimens were kept in a closed container stored in the laboratory under ambient conditions until the test day.

### 3.5. Compressive Strength Testing

Three sets of compressive strength tests were carried out (Table 3 and Table 4). The first set of tests were carried out after rest times of 2 h, 6 h, 12 h, 24 h, 30 h, and 36 h to determine the evolution of the early age strength of the different mixtures. The second set of tests were carried out after the samples were left to rest for the required time and then steam- or oven-cured. The third set of specimens were tested to determine the long-term temporal evolution of their compressive strength at ages of 1, 7, 28, 56, and 90 days of the ambient, steam-cured, and oven-cured specimens. 

### 3.6. Microstructure Analyses

#### 3.6.1. X-ray Diffraction (XRD)

Alkali-activated pastes (AAPs) were prepared and cured similarly to the corresponding AAM mixtures. Then, the AAP specimens were crushed into powder samples, and an X-ray diffraction (XRD) testing was conducted on each sample as well as on the corresponding raw FA using a Philips X-Pert diffractometer to determine the presence and evolution of the crystal phases. Paste specimens were used for the XRD analysis instead of mortars to exclude the participation of quartz crystals present in the sand particles, and thus relatively accurate results were obtained. To quantify the amorphous and crystalline phases, a 10 percent mass proportion of anatase titanium oxide (TiO_2_) was added to each sample powder. 

#### 3.6.2. Scanning Electron Microscopy (SEM) and Energy Dispersive X-ray Spectroscopy (EDS)

FA is a material predominantly composed of amorphous particles. The formed geopolymer gel resulting from the reaction of FA is mainly in an amorphous state. Thus, to complement the XRD data, SEM and EDS analyses were also conducted using an FEI Helios NanoLab 600 FIB/FESEM device with an SEM resolution (coincident point) of <1 nm to observe the microstructural development and to determine the elemental composition of the formed products.

## 4. Results and Discussion

### 4.1. Flow and Setting Properties of AAM

**Figure 3:**
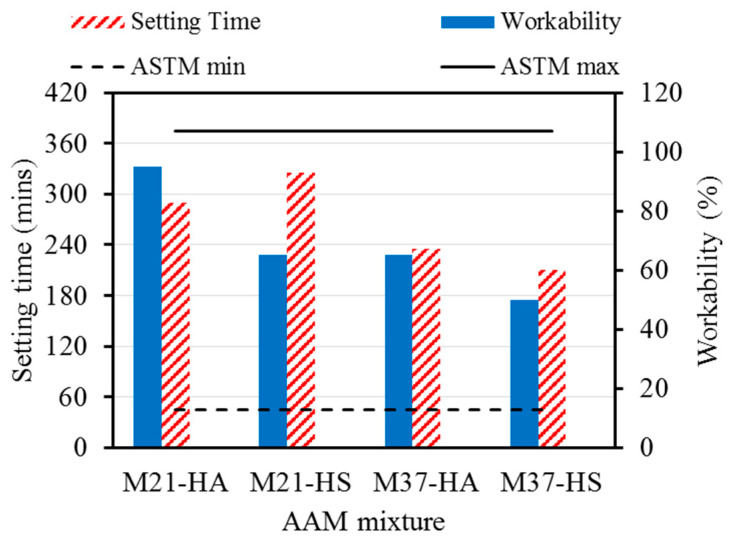
Fresh properties of AAM mixtures.

All the mixtures had good workability and an initial setting time that ranged from 210 to 325 min, respectively. All the setting times of the AAPs were within the specified limit values of ASTMmin and ASTMmax shown in Figure 3, respectively. The flow and setting times of the AAMs depended on the chemical composition of the precursor, such as the Si/Al ratio and Ca content, as well as that of the activator. Due to the high calcium content of FA37 compared to FA21, the M37 mixtures set faster than the M21 ones, with an average of 225 min for M37 and 310 min for M21. In addition, increasing the amount of SS in the HS mixtures compared to the HA mixtures led to poorer workability due to the high viscosity of the SS [2]. However, increasing the alkali content in the HA mixtures and the silicate content in the HS mixtures had a relatively similar effect on the initial setting time, where increasing either the alkali or the silicate content accelerated the initial strength development similarly.

### 4.2. Effects of Rest Time on the Short-Term Strength of AAM

#### 4.2.1. Ambient Curing Regime

**Figure 4:**
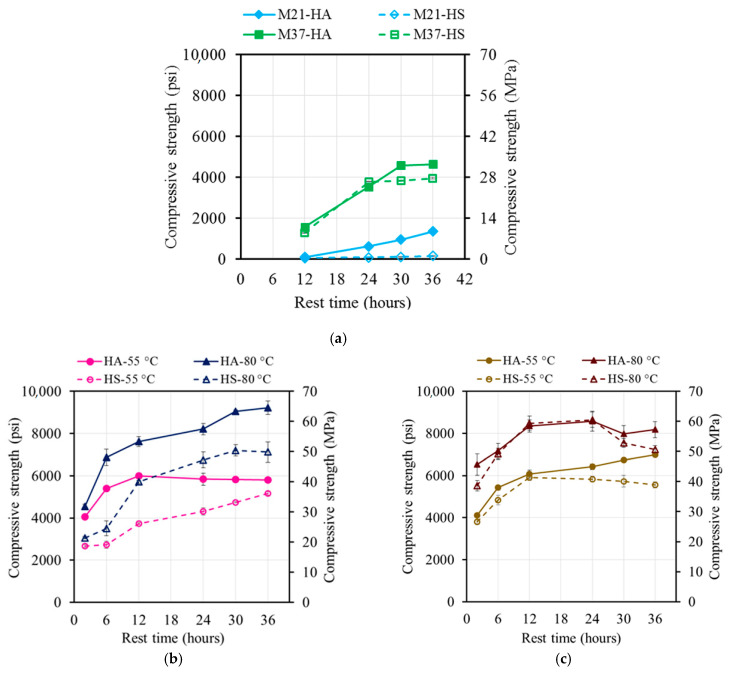
Effect of rest time on the compressive strength of (**a**) ambient-cured AAMs, (**b**) steam-cured M21, and (**c**) steam-cured M37.

The attempts to measure the compressive strengths at early ages of up to 6 h were not successful as the AAM specimens had not finally set yet or were not hard enough to be safely removed from their respective molds (Table 3, Figure 4a). The M37 mixtures displayed higher strengths than the M21 mixtures. At the ambient temperature and once the calcium in the FA came in contact with water, it dissolved, precipitated, nucleated, and grew into either calcium silicate hydrate (CSH) or calcium aluminate silicate hydrate (CASH), which were responsible for the strength development. Therefore, the higher the calcium content, the higher the strength.

After 12 h of rest time, the M37 mixtures developed an average compressive strength of 9.8 MPa (1430 psi) compared to 0.5 MPa (80 psi) for the M21 mixtures. Increasing the rest time increased the compressive strength until a rest time of 24 h and 30 h for M37-HS and M37-HA, which reached 31.8 MPa (4610 psi) and 26.6 MPa (3860 psi), respectively. The strengths of both the M21 mixtures, however, increased with time until a rest time of 36 h, reaching 9.3 MPa (1350 psi) and 1.10 MPa (160 psi) for M21-HA and M21-HS, respectively (Figure 4a). 

The observed difference in compressive strength between the HA and HS mixtures was primarily due to the Si/Al content. The HS mixture contained a relatively higher Si/Al or a lower concentration of Al compared to the HA mixture, which reduced the strength gain at early ages [8]. The high Si/Al ratio reduced the alkalinity by deprotonating hydrate silica molecules and consuming Na through the formation of aluminosilicate gel, thereby extending the early age of strength development [35,36]. The HA also provided a higher alkali content that accelerated the dissolution of the FA ions, such as Si, Ca, and Al, and enhanced the precipitation of the CASH, calcium sodium aluminate silicate hydrate (CNASH), and aluminosilicate gel at early ages [37]. Furthermore, HS mixtures provide more silica ions that initiate the formation of monomers, which is followed by the formation of polymers through geopolymerization, which requires, however, an elevated temperature to be triggered [4,29].

#### 4.2.2. Steam Curing Regime

**Figure 5:**
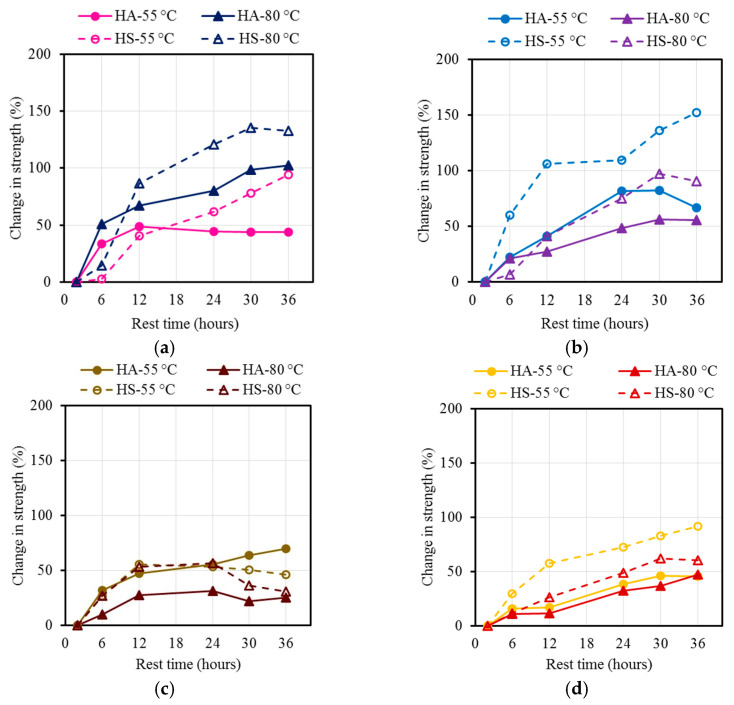
Percentage increase in strength of AAM relative to the compressive strength after 2 h of rest time of (**a**) steam-cured M21 mixtures, (**b**) oven-cured M21 mixtures, (**c**) steam-cured M37 mixtures, and (**d**) oven-cured M37 mixtures.

**Figure 6:**
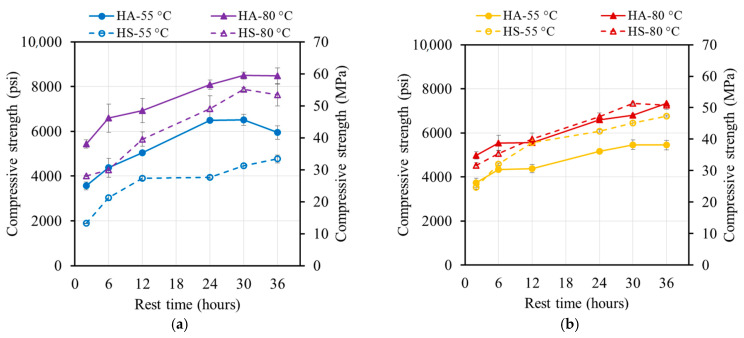
Effect of rest time on the compressive strength of oven-cured (**a**) M21 and (**b**) M37.

**Figure 7:**
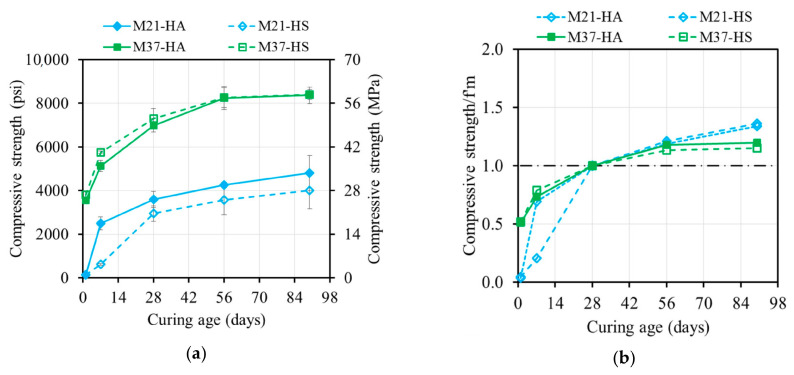
Ambient compressive strength development of AAM: (**a**) absolute value and (**b**) normalized f’_m_.

The steam-cured specimens at 80 °C displayed higher strength in comparison to those cured at 55 °C (Figure 4, Figure 5, Figure 6 and Figure 7). Increasing the temperature increased the energy available to break the Si-O covalent bond of the oligomers, thus allowing a fusion between two oligomers during the polycondensation phase of the geopolymerization. The rate and amount of the dissolved Si and Al ions from the FA particles in the presence of the alkaline ions were increased by increasing the curing temperature [10,11].

The strengths of the steam-cured mixtures ranged from 18.3 MPa (2660 psi) to 63.6 Mpa (5810 psi) for M21 and from 26.2 Mpa (5520 psi) to 59.6 Mpa (8650 psi) for M37 (Figure 4a,b), depending on the rest time, curing temperature, and activator constituents. For all the mixtures, significant increases in the compressive strengths occurred by increasing the rest time from 2 h to 12 h. The increases in the compressive strengths ranged from 40% to 86% and 28% to 56% for M21 and M37, respectively (Figure 4a,b and Figure 7). 

Increasing the rest time beyond 12 h resulted in different performances. Except for the M21-HA sample cured at 55 °C (131 °F), the compressive strengths of the M21 samples increased by an average of 115% after 12 h of rest time. Regardless of the rest time, the compressive strength remained constant. Furthermore, except for the M37-HA sample cured at 55 °C (131 °F), the compressive strengths of the M37 samples remained constant or decreased. For the M37-HA sample cured at 55 °C (131 °F), the compressive strength slightly increased with increasing the rest time.

The change in the performance of the different mixtures can be linked to the evolution of their strength during the rest time, which is linked to the chemical composition of each FA. The M21-HS and M21-HA samples reached approximately 0.7 Mpa (100 psi) at 30 h and 12 h, respectively, which is the minimum recommended compressive strength before starting steam curing for Portland-cement-based concrete [38]. When the rest time of the M21 samples was short, the specimens were exposed to an elevated temperature, which accelerated the precipitation of the aluminosilicate gel at early ages, which subsequently hindered the geopolymerization process and resulted in a lower compressive strength. However, a long rest period at room temperature before applying the elevated curing temperature allowed for a significant increase in fly ash dissolution and the formation of a continuous phase, which increased the homogeneity of the geopolymeric products [4]. In the case of the longer rest time of the M21 samples, however, adequate dissolution of the Si and Al ions occurred, and then the elevated temperature prompted the precipitation and formation of the aluminosilicate gel, which resulted in a higher compressive strength.

For a rest time for the M37 samples longer than 24 h, more CSH and/or CASH were formed at the ambient temperature due to the high calcium content of the FA37 sample, which subsequently limited the formation of the aluminosilicate gel at the elevated temperature, which resulted in a reduction in the strength. However, the presence of the calcium content at an elevated curing temperature was considered as a contaminant for the geopolymerization process [39,40,41,42,43], which also explained the higher compressive strength of the M21 samples compared with the M37 samples cured at high temperatures.

#### 4.2.3. Oven Curing Regime

The strengths of the oven-cured specimens ranged from 13.0 MPa (1890 psi) to 58.6 MPa (8490 psi) for M21 and from 25.7 MPa (3730 psi) to 50.7 MPa (7360 psi) for M37 (Figure 6), depending on the rest time, curing temperature, and activator constituents. For all the mixtures, significant increases in the compressive strengths, ranging from 56% to 136% and 37% to 83% for M21 and M37, respectively, (Figure 5 and Figure 6) occurred by increasing the rest time from 2 h to 30 h. The compressive strengths of the M21 samples were followed by a slight reduction in the compressive strength, except for M21-HS, whose compressive strength continuously increased, reaching 152% of the corresponding 2 h rest time compressive strength at 36 h of rest time.

Furthermore, except for the M37-HS sample cured at 80 °C (176 °F), the compressive strengths of the M37 samples remained constant or increased. For the M37-HA sample cured at 55 °C (131 °F), the compressive strength slightly decreased with increasing the rest time.

In the case of the oven curing regime and M37, the compressive strength increased as the compressive strength increased. The longer rest time and the presence of a relatively high calcium content allowed for the initiation of the formation of hydration products, i.e., CSH and CASH, at the ambient temperature at early ages, which resulted in increasing the overall compressive strength. However, the overall compressive strengths of all the mixtures were lower than those of the steam-cured specimens, where the relatively high calcium content interfered with the formation of the aluminosilicate gel and resulted in a decrease in the compressive strength [39]. However, in the case of the steam curing regime and M37, the presence of an environment of moisture during curing facilitated both the hydration and geopolymerization processes, which subsequently improved the overall compressive strength. 

This section of the study displayed that for the M21 mixtures synthesized using a low calcium content, both curing regimes yielded very close strength values. The M37 mixtures synthesized using a high calcium content and steam curing at 80 °C (176 °F) consistently yielded an approximately 17% higher strength. Similarly, the 55 °C (131 °F) steam-cured M37-HA sample displayed a 27% higher strength than the corresponding oven-cured mixture. However, the M37-HS sample steam-cured at 55 °C (131 °F) was 17% weaker than the corresponding oven-cured mixture. Furthermore, in no case did a rest time of 12 h result in a reduction in the compressive strength. Most mixtures reached from 72% to 100% of their peak strengths when they had a rest time of 12 h. Therefore, considering practicality, 12 h was selected as the optimum rest time for all the mixtures used in the remainder of this study. However, if a prolonged rest time is allowed in real-world applications, a rest time of between 24 and 30 h would result in the highest possible compressive strength.

### 4.3. Long-Term Strength Development of AAM

#### 4.3.1. Ambient-Cured Specimens

The ambient-cured specimens demonstrated increasing strength over time (Table 4 and Figure 7) due to the formation of different products at varying rates. The M37 mixtures exhibited an approximately 187% higher strength than the M21 ones on average. The higher calcium content in the M37 mixtures resulted in more hydration products such as CSH and CASH, leading to a stronger compressive strength at ambient temperature. Conversely, the M21 mixtures, with their high silica and alumina contents, required an elevated temperature for geopolymerization, resulting in a lower compressive strength when ambient curing was used.

The compressive strengths of the M37 mixtures reached 0.51 times the strength at 28 days (f’m) after one day of curing. The strength continued to increase up to 56 days, with no significant development beyond that. Both the M37 and M21 samples developed strength at similar rates due to the presence of Ca-rich products and the governing hydration reaction. The strength development process was determined by the availability of Ca ions, while the extra silica and sodium species had minimal impact. The strength of the M37 samples increased rapidly up to 28 days and reached an average of 118% of f’m at 90 days.

The M21 mixtures initially had low compressive strengths after one day but significantly increased over time. The lower calcium content in the precursor FA21 led to the formation of fewer hydration products during hardening. The M21-HA sample, with its higher alkali content, exhibited relatively rapid strength development within a week, reaching 69% of f’m after one week and 134% of f’m at 90 days. 

The M21-HS sample demonstrated lower strength development in the first two weeks but progressively increased, reaching 20.3 MPa (2950 psi). Subsequently, it followed a similar rate of increase as M21-HA, attaining 136% of f’m at 90 days. The slower polycondensation rate of polysialate-disioloxo in the M21-HS sample, which was caused by the higher silica concentration in the activator, contributed to its lower strength development compared to the M21-HA sample at the early ages.

#### 4.3.2. Thermally Cured Specimens

**Figure 8:**
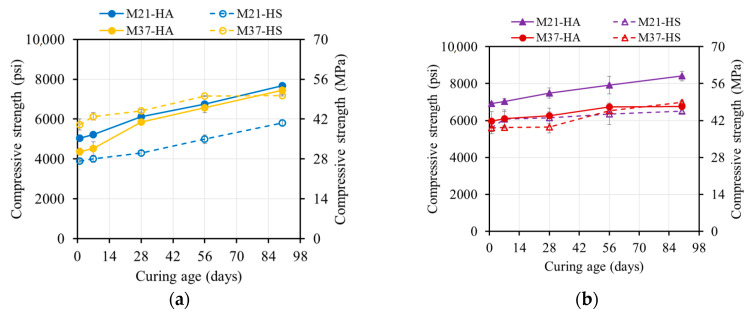
Compressive strength evolution of oven-cured specimens cured at (**a**) 55 °C (131 °F) and (**b**) 80 °C (176 °F).

**Figure 9:**
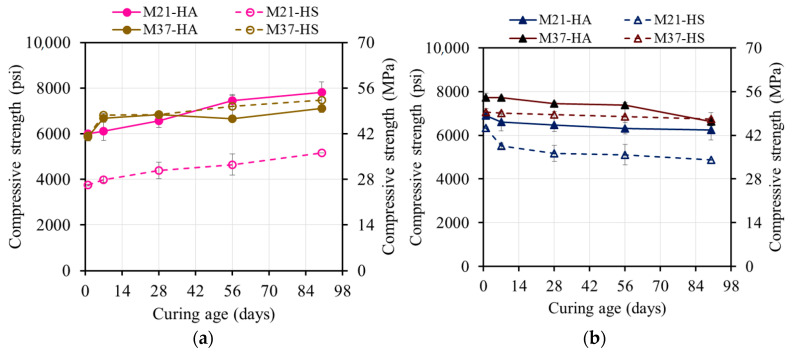
Compressive strength evolution of steam-cured specimens cured at (**a**) 55 °C (131°F) and (**b**) 80 °C (131 °F).

Elevated temperatures had a significant influence on the geopolymerization process, since the reaction kinetics required a certain amount of heat in order to occur. However, the reaction did not stop at the end of the thermal curing process (Figure 8 and Figure 9).

##### Oven-Cured Strength Development

The 55 °C (131 °F) oven-cured one-day compressive strengths for mixtures M21 and M37 were 30.8 and 34.3 MPa (4470 and 4970 psi), respectively. Increasing the curing temperature to 80 °C (176 °F) resulted in strength increases to 43.3 and 39.0 MPa (6270 and 5790 psi), corresponding to strength increases of 42% and 15%, respectively (Figure 8). A higher curing temperature accelerated the formation and growth of geopolymer products, leading to a relatively higher strength.

The strength of the M21 and M37 samples increased over time, with a faster rate at 55 °C (131 °F). The 90-day compressive strength values at 55 °C (131 °F) and 80 °C (176 °F) were 45–70% and 10–15% higher, respectively, compared to the one-day compressive strengths. These values reached 40.1–57.2 MPa (5820–8300 psi) and 46.6–58.0 Mpa (6760–8410 psi).

At 80 °C (176 °F), the rate of the strength increase was limited due to the reacted products covering the surfaces of the FA particles. This blocked the dissolution sites and reduced the formation of geopolymer products. In contrast, the specimens cured at 55 °C (131°F) had fewer formed products, exposing more surfaces and increasing the number of dissolution sites. Over time, dissolved species from the FA particles condensed and formed new products, resulting in a strong and stable gel structure and a higher strength [4,18].

##### Steam-Cured Strength Development

The strength of the steam-cured specimens increased over time, with a higher rate of increase for the 55 °C (131 °F) steam-cured specimens. The 90-day compressive strength values at 55 °C (131 °F) were 21–38% higher compared to the one-day compressive strengths, reaching values of 35.6–53.8 MPa (5160–7810 psi) (Figure 9). However, for the specimens steam-cured at 80 °C (176 °F), the strength decreased by 5–23%, reaching 33.6–46.4 MPa (4870–6730 psi) (Figure 9b).

In the case of the 80 °C (176 °F) oven curing, the rapid rate of reaction led to quick polycondensation between oligomers. This resulted in the geopolymeric gel forming rapidly and surrounding most of the unreacted FA particles, reducing dissolution sites and potentially blocking the reaction process. Additionally, the high relative humidity during steam curing (95%) followed by storage in lower-humidity ambient conditions caused moisture loss in the AAM specimens, creating voids.

For the specimens cured at 55 °C (131 °F), dissolution continued, and the voids were progressively filled with newly formed products, leading to an increase in strength. However, for the specimens cured at 80 °C (176 °F), no further products were formed, and the voids remained unfilled, weakening the structure of the geopolymeric gel and resulting in strength regression.

#### 4.3.3. Strength of Ambient-Cured vs. Thermally Cured Specimens

**Figure 10:**
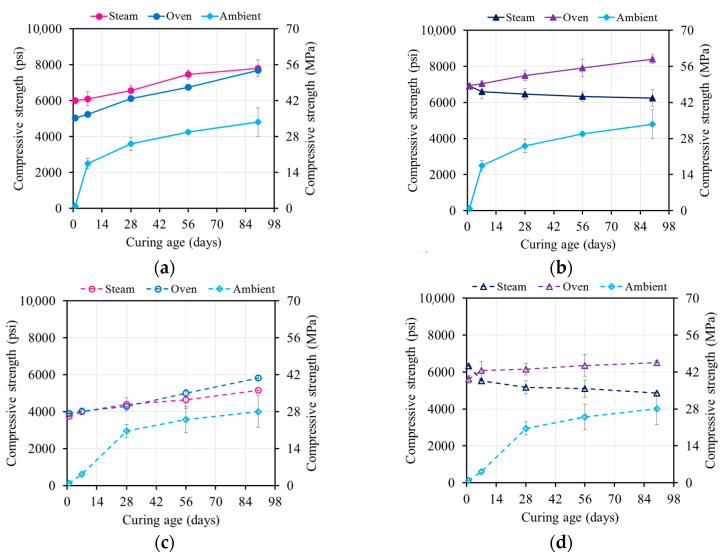
Comparison between ambient curing, oven, and steam curing for: (**a**) M21-HA at 55 °C (131 °F), (**b**) M21-HA at 80 °C (176 °F), (**c**) M21-HS at 55 °C (131 °F), and (**d**) M21-HS at 80 °C (176 °F).

**Figure 11:**
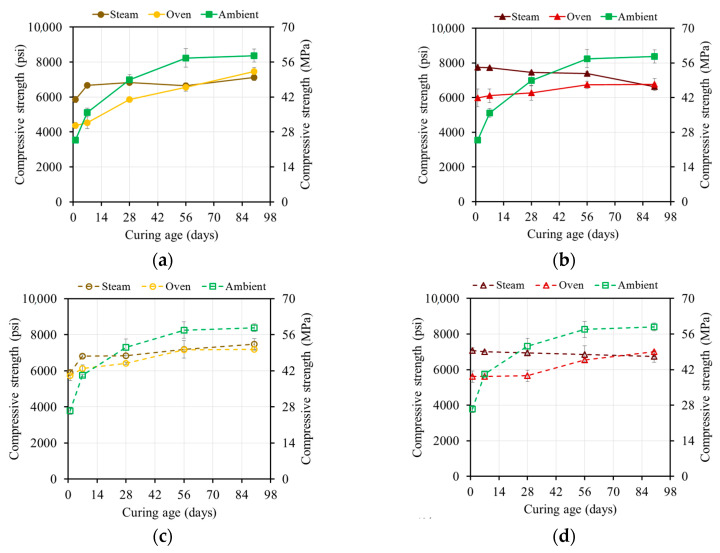
Comparison between ambient curing, oven curing, and steam curing for (**a**) M37-HA at 55 °C (131 °F), (**b**) M37-HA at 80 °C (176 °F), (**c**) M37-HS at 55 °C (131 °F), and (**d**) M37-HS at 80 °C (176 °F).

**Figure 12:**
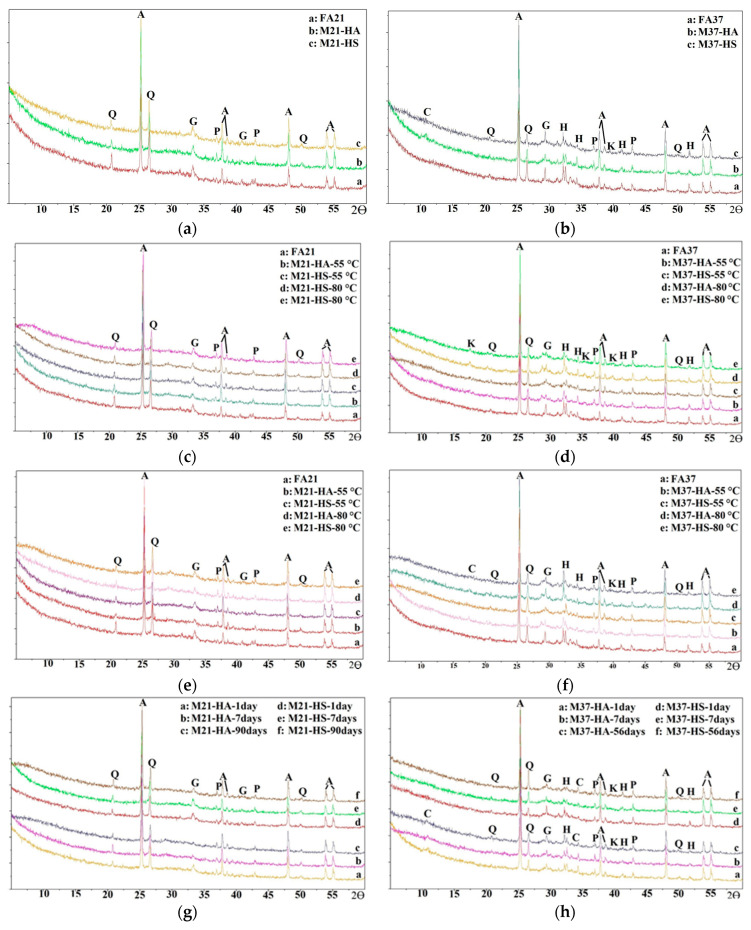
XRD patterns of unreacted FAs and AAPs: (**a**) P21 mixtures ambient-cured for one day, (**b**) P37 mixtures ambient-cured for one day, (**c**) oven-cured P21 mixtures, (**d**) oven-cured P37 mixtures, (**e**) steam-cured P21 mixtures, (**f**) steam-cured P37 mixtures, (**g**) stored and tested M21 mixtures, and (**h**) stored and tested M37 mixtures.

Figure 10 and Figure 11 summarize the effect of the three curing regimes on the different mixtures. Oven and steam curing at either 55 °C or 80 °C (131 °F or 176 °F) resulted in higher one-day compressive strengths compared to ambient curing (Figure 8, Figure 9, Figure 10, Figure 11 and Figure 12). Both steam and oven curing showed similar one-day compressive strengths. The 90-day compressive strengths for the oven- and steam-cured specimens were 30–63% and 60–75% higher than those of the ambient-cured specimens, respectively. The accelerated geopolymerization process in the presence of heat contributed to the higher strength. The specimens that were oven- and steam-cured at 55 °C (131 °F) exhibited similar strengths, while the specimens that were oven-cured at 80 °C (176 °F) displayed a 32–35% higher strength compared to the steam-cured ones, which lost strength over time.

The one-day strength of the oven-cured M37 mixtures showed a similar development to the M21 mixtures due to the hindered leaching properties of calcium at elevated temperatures. The one-day compressive strength of the steam-cured M37 mixtures was 56–126% higher than that of the ambient-cured specimens and 3–34% higher than that of the oven-cured specimens. The moisture provided by the steam curing was beneficial for the reaction, leading to a higher strength at the end of the curing regime. However, the 90-day strength of the oven- or steam-cured M37 mixtures was approximately the same and 10–15% lower than that of the ambient-cured specimens, which was related to the type of formed products.

### 4.4. Microstructure and Nanostructure Analysis

#### 4.4.1. X-ray Diffraction Analysis

Figure 12 shows the results of the XRD measurements conducted on the raw FAs and paste powder from each mixture cured in ambient conditions for one day. The main crystal phases present in the FA37 sample were anatase (hereinafter, A), periclase (hereinafter, P), quartz (hereinafter, Q), gehlenite (hereinafter, G), and hatrurite (hereinafter, H), while those in the FA21 sample were A, Q, G, and P, and these were analyzed based on the findings of previous studies [44,45,46,47,48]. The presence of a crystal was mainly due to its addition as an internal component for crystal-phase quantification purposes (Table 5). The XRD patterns of the M21 mixtures (Figure 12a) did not show any significant change compared to those of the raw FA, which confirmed the low early ambient compressive strengths. The XRD patterns of the M37 samples (Figure 12b) showed a reduction in the peak intensities of Q, G, and H, indicating the dissolution of these compounds, which was confirmed by the reduction in the crystalline content of the AAP samples compared with their raw FA (Table 5). The XRD patterns displayed new small sharp peaks corresponding to the formation of calcium silicate hydrate (CSH) (hereinafter, C) and katoite (hereinafter, K), which is mainly composed of calcium aluminosilicate hydrate products (CASH), responsible for the early high strength of the M37 mixtures cured at ambient conditions (Figure 7). The reduction in Q and H was higher in the case of M37-HS compared to M37-HA, indicating that more geo-polymerization and hydration reactions took place. However, there was more dissolution of G and a higher peak of C for M37-HA compared to M37-HS. These changes in Q, G, H, and C balanced each other and can explain the small difference in the compressive strength between M37-HA and M37-HS. It is worth mentioning that the noise-like appearance of the CSH peak in the EDS test may stem from various experimental factors, such as the instrument settings and material characteristics, while the discrete and consistent appearance of the CASH peak suggests its robust presence in the sample, possibly due to factors like the concentration, crystal structure, or sample purity.

The XRD patterns of the oven-cured mixtures are shown in Figure 12c,d. In the case of the M21 mixtures, there was a reduction in the peak intensity of Q and G, indicating a partial dissolution of these compounds. The reduction in the peak was more significant in the case of M21-HA, which contributed to the higher strength of M21-HA compared to M21-HS. There was also a noticeable broad hump forming at 28°, 30°, and 41°, which indicated the formation of an amorphous product from the geopolymeric reaction. In addition, a wider hump was noticed in the XRD patterns of the mixtures cured at 80 °C than in the patterns of those cured at 55 °C (131 °F), since more product was formed at the higher temperature, as explained earlier. A much broader hump implies a much denser gel structure and, hence, a higher strength, which explains the difference in the strength of the mixtures cured at these two temperatures (Figure 10). The crystalline content decreased from 13% in FA21 to 4–7% in the M21 mixtures, indicating that more amorphous products were formed (Table 5). However, no new peaks were formed, indicating that there were no new crystalline compounds formed. 

For the M37 oven-cured mixtures (Figure 12d), the formation of small peaks at 17° and 28° indicated the presence of CASH products, i.e., K. The reaming peaks in the M37 samples corresponded to the crystal phases present in the raw FA37. Therefore, the strength development of the oven-cured M37 samples, as observed in Figure 8, mainly depended on the growth of CASH products. The absence of any CSH formation in the XRD results occurred due to the fact that elevated temperatures hindered the leaching of calcium in the FA [49]. 

The XRD patterns of the steam-cured M21 mixtures (Figure 12e,f) were similar to those of the oven-cured ones but with wider humps observed at 28°–30° and 41° for the steam-cured specimens, which explains the slightly higher strength of the steam-cured mixtures compared to the oven-cured ones. As explained earlier, a wider hump represents the formation of a denser amorphous gel. 

In the case of the M37 mixtures, in addition to the broad hump occurring at 28°–30°, new peaks were formed at 38°, which were due to K crystals forming (CASH) (Figure 12f). The sharper peaks of the K crystals with their high intensities and broader humps indicated amorphous compounds, which were observed in the mixtures cured at 80 °C (176 °F) and not in those cured at 55 °C (176 °F), which explains the higher compressive strength of these mixtures compared to those cured at a lower temperature. The XRD patterns of the ambient-cured specimens examined for the long-term study showed that there was a slight reduction in the main peaks over the time due to the continuous dissolution of the original compounds of the raw FA that participated in the formation of products in the amorphous phase (Figure 12g,h).

#### 4.4.2. SEM and EDS Analysis

**Figure 13:**
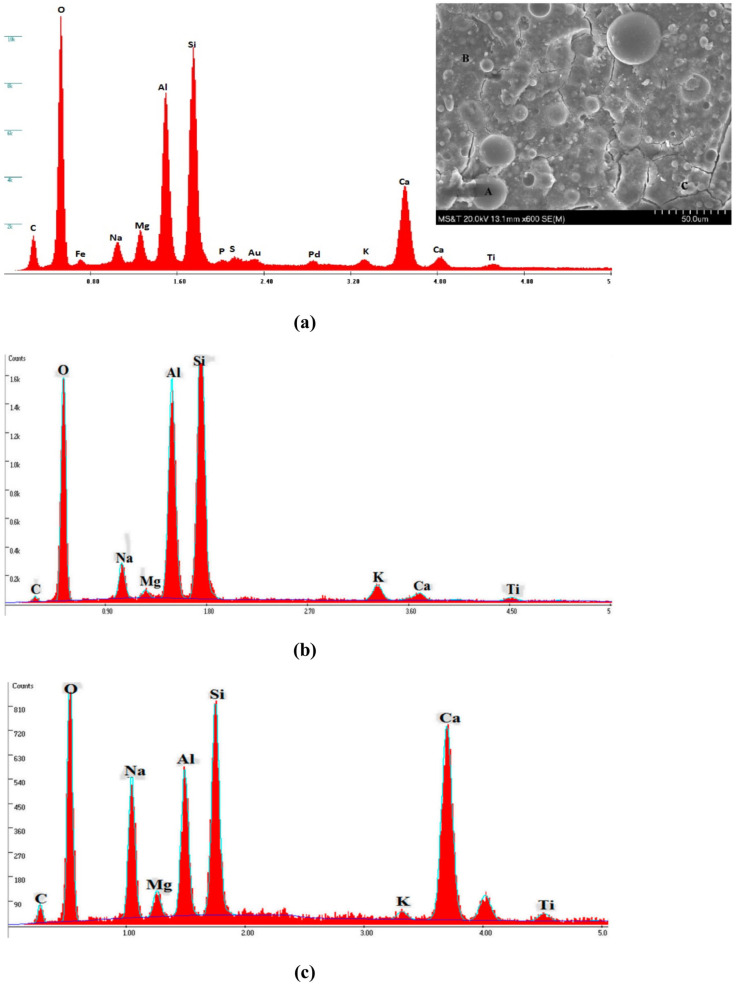
EDS results of: (**a**) Unreacted FA21, (**b**) Reacted P21 FA particle “A”, and (**c**) Reacted P21 FA particle “B”.

The SEM images of P21 samples that were steam-cured at 80 °C (176 °F) are shown in Figure 13a. Three products were obtained in the P21 samples, which were labeled as A, B, and C (Figure 13). A represents partially unreacted FA particles and spherical shapes that varied in sizes (Figure 13a). The EDS results showed that the main elements in the unreacted FA21 particles were O, Si, Al, and Ca, with the presence of Na, Mg, g, and Fe in small quantities (Figure 13a).

After the FA came into contact with water and liquid activators, dissolved species from these FA particles reacted with available dissolved ions from the activators and led to the formation of a gel that linked all the separate FA particles together (Figure 13a). There was the formation of a light layer around each FA particle, representing the resulting products formed during the geopolymerization and hydration processes.

Two different areas, B and C, were observed and analyzed using EDS. B represents the area of a formed geopolymerization product (NASH) (Figure 13b). The EDS results of B showed a very low intensity of Ca relative to the intensities of Si, Al, Na, and O, which is an indication of the formation of NASH. Furthermore, the predominant elements present in area “C”, the formed product gel area, were Ca, Na, Si, Al, and O (CNASH) (Figure 13c). CNASH yields a fast strength development if cured at a high temperature. In addition, the presence of many partially reacted FA particles fully surrounded by the formed aluminosilicate gel was due to the faster growth of the formed products, since this paste sample was originally steam-cured at 80 °C (176 °F). It is evident that further dissolution of elements would be hindered and, thus, as a consequence, low or no strength development would occur over time. This occurred in the samples that were steam-cured at 80 °C (176 °F), as was the case with all the AAM specimens.

## 5. Conclusions

Four AAM mixtures with varying mixing proportions were synthesized using two different types of class C FA, (FA37 and FA21). These AAM mixtures were subjected to different rest times ranging from 2 h to 36 h prior to being ambient-, oven-, or steam-cured. The steam- and oven-curing processes were conducted at 55 °C (131 °F) and 80 °C (176 °F). The short- and long-term compressive strengths of the samples were studied. Furthermore, the microstructural characteristics of the samples were studied to examine the reaction products. From the obtained results, it was concluded that:

- Each mixture showed an optimal rest time ranging from 12 to 30 h, beyond which the strength decreased. A twelve-hour rest time led to a significant 46% increase in the compressive strength compared to a two-hour rest time.
- The ambient-cured M37 mixtures with a higher calcium content had higher strengths, reaching approximately 49.3 MPa (7160 psi) at 28 days. The ambient-cured M21 mixtures with a lower calcium content showed lower initial strengths but significant increases over time, reaching 24.8 MPa (3600 psi) at 28 days and about 35% higher strengths at 90 days.
- Thermal curing, especially at 80 °C (176 °F), resulted in rapid early strength development. However, for the curing conducted at 55 °C (131 °F), a significant strength increase occurred between 1 and 90 days, with no differences observed between the steam and oven curing. Steam curing at 80 °C (176 °F) led to a reduction of up to 23% due to self-desiccation.
- The ambient-cured M37 mixtures had higher strengths than the thermally cured ones at 56 and 90 days, which were up to 26% higher. the ambient-cured M21 mixtures had the lowest strength compared to those subjected to thermal curing due to their relatively low calcium content.
- XRD analysis showed hydration product formation in the M37 mixtures, while FA21 compound peaks decreased in the M21 mixtures.
- The EDS spectra indicated partially unreacted FA particles, showing the effect of curing on FA dissolution and geopolymer product formation that were responsible for the strength development.

## Figures and Tables

**Table 1:** X-ray fluorescence of FAs.

FA	CaO	Al_2_O_3_	SiO_2_	MgO	Fe_2_O_3_	Na_2_O	TiO_2_	P_2_O_5_	K_2_O	LOI
FA37	36.9	14.0	36.9	4.80	3.52	1.62	0.87	0.70	0.62	0.50
FA21	21.1	20.1	43.9	4.29	4.96	2.87	1.36	0.51	0.70	0.40

**Table 2:** Mix design ratio and quantities in kg/m^3^ (lb/yd^3^).

Mix Name	HA ^a^	HS ^b^
Alk/FA	0.300	0.275
W/FA	0.380	0.400
SS/SH	1.000	2.000
SH	82 (138)	49 (83)
SS	82 (138)	98 (165)
Water	113 (190)	132 (222)
Sand	1500 (2528)	1470 (2478)
FA	545 (918)	535 (902)

^a^ HA: high-alkali mixture, ^b^ HS: high-silicate mixture.

**Table 3:** Summary of short-term compressive strength development.

Mix	Rest Time	Compressive Strength									
	(hrs)	Ambient *		Oven—55 °C		Oven—80 °C		Steam—55 °C		Steam—80 °C	
		psi	MPa	psi	MPa	psi	MPa	psi	MPa	psi	MPa
M21-HA	2	-	-	3570	24.6	5440	37.5	4040	27.9	4560	31.4
	6	-	-	4370	30.1	6590	45.4	5400	37.2	6870	47.4
	12	110	0.70	5040	34.7	6930	47.8	6010	41.4	7620	52.5
	24	630	4.30	6480	44.7	8090	55.8	5850	40.3	7860	54.2
	30	950	6.60	6510	44.9	8490	58.6	5820	40.1	9050	62.4
	36	1350	9.30	5950	41.0	8470	58.4	5810	40.1	9220	63.6
M21-HS	2	-	-	1890	13.0	4000	27.6	2660	18.3	3060	21.1
	6	-	-	3020	20.8	4270	29.4	2730	18.8	3510	24.2
	12	50	0.30	3900	26.9	5650	39.0	3740	25.8	5710	39.4
	24	86	0.60	3950	27.3	7010	48.4	4300	29.7	6750	46.6
	30	110	0.80	4460	30.8	7890	54.4	4740	32.7	7200	49.7
	36	160	1.10	4760	32.8	7630	52.6	5160	35.6	7120	49.1
M37-HA	2	-	-	3730	25.7	4980	34.3	4120	28.4	6520	45.0
	6	-	-	4330	29.8	5530	38.1	5430	37.4	7170	49.4
	12	1570	10.8	4380	30.2	5570	38.4	6070	41.9	8340	57.5
	24	3540	24.4	5170	35.7	6600	45.5	6420	44.2	8590	59.2
	30	4580	31.6	5460	37.7	6800	46.9	6740	46.4	7970	54.9
	36	4640	32.0	5450	37.5	7330	50.5	6990	48.2	8180	56.4
M37-HS	2	-	-	3520	24.3	4520	31.2	3800	26.2	5520	38.1
	6	-	-	4580	31.6	5060	34.9	4830	33.3	7030	48.5
	12	1280	8.80	5560	38.3	5730	39.5	5910	40.8	8480	58.4
	24	3790	26.1	6080	41.9	6740	46.5	5830	40.2	8650	59.6
	30	3830	26.4	6450	44.5	7350	50.7	5730	39.5	7550	52.0
	36	3960	27.3	6760	46.6	7250	50.0	5560	38.3	7230	49.9

* Immediately after the required rest time.

**Table 4:** Long-term compressive strength.

Mix	Age	Compressive Strength									
	(days)	Ambient		Oven—55 °C		Oven—80 °C		Steam—55 °C		Steam—80 °C	
		psi	MPa	psi	MPa	psi	MPa	psi	MPa	psi	MPa
M21-HA	1	130	0.90	5040	34.7	6930	47.8	6010	41.4	6910	47.7
	7	2490	17.2	5230	36.0	7040	48.5	6100	42.1	6600	45.5
	28	3590	24.8	6120	42.2	7490	51.6	6560	45.2	6470	44.6
	56	4260	29.3	6750	46.5	7920	54.6	7460	51.4	6320	43.6
	90	4800	33.1	7680	52.9	8410	58.0	7810	53.8	6250	43.1
M21-HS	1	140	1.00	3900	26.9	5610	38.7	3740	25.8	6340	43.7
	7	610	4.20	4010	27.7	6090	42.0	3990	27.5	5520	38.1
	28	2950	20.3	4290	29.6	6160	42.5	4390	30.3	5180	35.7
	56	3570	24.6	4990	34.4	6360	43.9	4640	32.0	5110	35.2
	90	4010	27.6	5820	40.1	6500	44.8	5160	35.6	4870	33.6
M37-HA	1	3539	24.4	4380	30.2	5970	41.2	5860	40.4	7730	53.3
	7	5120	35.3	4520	31.2	6110	42.1	6680	46.0	7720	53.2
	28	6990	48.2	5850	40.3	6260	43.2	6830	47.1	7460	51.4
	56	8240	56.8	6560	45.3	6730	46.4	6650	45.9	7390	51.0
	90	8370	57.7	7460	51.4	6760	46.6	7120	49.1	6630	45.7
M37-HS	1	3790	26.1	5730	39.5	5610	38.7	5910	40.8	7070	48.8
	7	5750	39.6	6140	42.3	5620	38.7	6810	47.0	7010	48.4
	28	7310	50.4	6420	44.3	5650	39.0	6840	47.1	6940	47.9
	56	8250	56.9	7160	49.4	6540	45.1	7190	49.6	6850	47.2
	90	8390	57.9	7190	49.5	7000	48.3	7480	51.6	6730	46.4

**Table 5:** Measured crystalline content (%).

Mix	Days	Ambient	Oven—55 °C	Oven—80 °C	Steam—55 °C	Steam—80 °C
FA21	12.9					
M21-HA	1	7.6	7.1	6.1	5.8	5.4
	7	6.1	7.5	8.4	6.7	6.6
	90	4.1	5.7	5.4	4.1	5.3
M21-HS	1	7.3	6.0	5.7	6.3	6.7
	7	8.8	6.4	7.1	7.3	7.9
	90	6.2	7.3	7.8	5.6	7.3
FA37	15.9					
M37-HA	1	6.9	8.0	8.2	7.2	7.2
	7	6.8	8.7	6.2	9.7	8.2
	56	6.4	6.8	7.8	6.1	7.0
M37-HS	1	6.1	5.8	5.2	5.9	6.2
	7	5.2	7.8	8.4	7.6	6.2
	56	7.6	7.2	5.4	6.1	6.3

## Data Availability

Data are contained within the article.
